# Platform independent protein-based cell-of-origin subtyping of diffuse large B-cell lymphoma in formalin-fixed paraffin-embedded tissue

**DOI:** 10.1038/s41598-020-64212-z

**Published:** 2020-05-12

**Authors:** Jörg Reinders, Michael Altenbuchinger, Katharina Limm, Philipp Schwarzfischer, Tamara Scheidt, Lisa Strasser, Julia Richter, Monika Szczepanowski, Christian G. Huber, Wolfram Klapper, Rainer Spang, Peter J. Oefner

**Affiliations:** 1 Chair and Institute of Functional Genomics, University of Regensburg, 93053 Regensburg, Germany; 2 Present Address: Analytical Chemistry Support Unit, Leibniz Research Centre for Working Environment and Human Factors, 44139 Dortmund, Germany; 3 Chair of Statistical Bioinformatics, Institute of Functional Genomics, University of Regensburg, 93053 Regensburg, Germany; 4 Department of Biosciences, Bioanalytical Research Labs, and Cancer Cluster Salzburg, University of Salzburg, 5020 Salzburg, Austria; 5 Department of Pathology, Hematopathology Section and Lymph Node Registry, University Hospital Schleswig-Holstein, Campus Kiel/Christian-Albrecht University, 24106 Kiel, Germany

**Keywords:** Biomarkers, Oncology

## Abstract

Diffuse large B-cell lymphoma (DLBCL) is commonly classified by gene expression profiling according to its cell of origin (COO) into activated B-cell (ABC)-like and germinal center B-cell (GCB)-like subgroups. Here we report the application of label-free nano-liquid chromatography - Sequential Window Acquisition of all THeoretical fragment-ion spectra – mass spectrometry (nanoLC-SWATH-MS) to the COO classification of DLBCL in formalin-fixed paraffin-embedded (FFPE) tissue. To generate a protein signature capable of predicting Affymetrix-based GCB scores, the summed log_2_-transformed fragment ion intensities of 780 proteins quantified in a training set of 42 DLBCL cases were used as independent variables in a penalized zero-sum elastic net regression model with variable selection. The eight-protein signature obtained showed an excellent correlation (r = 0.873) between predicted and true GCB scores and yielded only 9 (21.4%) minor discrepancies between the three classifications: ABC, GCB, and unclassified. The robustness of the model was validated successfully in two independent cohorts of 42 and 31 DLBCL cases, the latter cohort comprising only patients aged >75 years, with Pearson correlation coefficients of 0.846 and 0.815, respectively, between predicted and NanoString nCounter based GCB scores. We further show that the 8-protein signature is directly transferable to both a triple quadrupole and a Q Exactive quadrupole-Orbitrap mass spectrometer, thus obviating the need for proprietary instrumentation and reagents. This method may therefore be used for robust and competitive classification of DLBCLs on the protein level.

## Introduction

Diffuse large B-cell lymphoma (DLBCLs) is commonly grouped into three distinct molecular subtypes based on the putative cell of origin (COO): the activated B-cell-like (ABC), the germinal B-cell-like (GCB), and the unclassifiable subtype as defined by array-based gene expression profiling^1^. Numerous studies have confirmed the distinct differences in biology and clinical behavior of these subtypes and, therefore, determination of COO has become an integral component in the diagnosis of DLBCL, providing a basis for targeted-treatment stratification^2^. GCB-like DLBCL has shown consistently better response to treatment with cyclophosphamide, doxorubicin, vincristine, and prednisone (CHOP) without and with the addition of rituximab (R-CHOP). However, the requirement for high quality tumor RNA from snap frozen DLBCL biopsies represented a significant obstacle to the routine determination of COO. To meet the needs of standard clinical practice, which prefers the collection of formalin-fixed paraffin-embedded (FFPE) tissue, alternative approaches have been pursued. These focused initially on immunohistochemical approaches examining the expression of two to four protein markers^3^. The most widely adopted approach has been the “Hans” classifier, which exploits antibody staining of CD10, BCL6, and IRF4, to group DLBCLs into GCB- and non-GCB-like DLBCLs^4^. While initial studies reported good concordance between COO immunophenotyping and microarray-based COO profiling, application of the “Hans” classifier to a blinded set of 949 biopsies collected in the RICOVER-60 trial failed to show prognostic significance both in the entire cohort as well as in the subgroups receiving CHOP with and without rituximab^5^. In 2011, Rimsza *et al*.^6^ reported the use of a quantitative nuclease protection assay to analyze expression levels of 12 genes including *CD10, LRMP, CCND2, ITPKB, PIM1, IL16, IRF4, FUT8, BCL6, LMO2, CD39*, and *MYBL1*, in DLBCL FFPE tissues. Predictor scores obtained for these 12 genes and an Affymetrix-based signature of 187 genes showed overall good agreement with a linear regression *R*-scale value of 0.89 and *P* < 0.0001, with only four major discrepancies (3 ABC were called as GCB and one GCB as ABC) between the two methods. In 2013, we were the first^7^ to report the use of the NanoString nCounter gene expression system, which allows the multiplexed capture and direct counting of individual mRNA transcripts with color-coded probe pairs without enzymatic reactions^8^, for the molecular classification of DLBCL using FFPE-derived RNA. Contrary to the “Hans” classifier, which yielded three major misclassifications (2 GCB classified as non-GCB and 1 ABC as GCB) out of 23 (13%) DLBCL specimens examined both by immunostaining and microarray-based typing of snap-frozen tissue, nCounter-based expression analysis of 20 genes, which had been previously found by microarray-based analysis to differentiate ABC- from GCB-like DLBCLs, yielded only minor discrepancies, *i.e*., the switching between the unclassified and ABC or the unclassified and GCB labels in 5 of 31 (16%) specimens analyzed. Since others could subsequently confirm these promising results^9–11^, with >95% concordance of COO assignments between laboratories, the NanoString nCounter system has become the new benchmark of DLBCL COO determination in clinical trials. However, dependence on a single provider of instrumentation, reagents and software, has continued to spur the development of novel approaches to COO determination in FFPE material such as massive parallel quantitative reverse transcription PCR^12^ and shotgun liquid chromatography-mass spectrometry^13^. In addition, there is a continued need for the identification of both COO subtype-specific as well as COO independent biomarkers for risk stratification of DLBCL patients undergoing immunochemotherapy. The presence of *BCL2* gain/amplification, for instance, was reported to be significantly associated with poor outcome in ABC-like DLBCL, while *BCL2* translocation predicted poor outcome in GCB-like DLBCL^14^. Differences in the expression of various microRNAs, on the other hand, were associated with survival in R-CHOP-treated patients independently of COO^15^.

Here we report the application of Sequential Window Acquisition of all THeoretical fragment-ion spectra mass spectrometry (SWATH-MS), which allows for the precise quantification of peptides without the need for differential stable-isotope labeling^16^, to the COO classification of DLBCL. SWATH-MS combines the advantages of untargeted shotgun proteomics by covering hundreds to thousands of protein groups in a single analysis and selected reaction monitoring mass spectrometry by yielding highly reproducible and consistent data. This was recently demonstrated for a comparative analysis of cryopreserved and FFPE tissue sections of Burkitt’s lymphoma and DLBCL with more than 90% of the proteins that differed significantly between these two lymphomas showing the same direction of regulation regardless of tissue preservation^17^. Using a reference point insensitive regularized regression method with variable selection^18,19^, we report here the identification of an eight-protein signature that yields not only excellent correlation with GCB predictor scores derived from both Affymetrix GeneChip and NanoString nCounter gene expression data but also carries the advantage of being transferable to other analytical platforms as long as the abundance of the signature proteins can be measured within the linear dynamic ranges of the respective methods.

## Specimens and Methods

### Specimens

All specimens were collected and analysed by an approved experimental protocol by the Molecular Mechanisms of Malignant Lymphoma (MMML) consortium, after the patients had given their informed consent, and analysed in accordance with the guidelines and regulations of the ethics board of the Medical Faculty, University of Kiel, for the use of archival tissue (D447/10). The training cohort comprised 42 FFPE tissue sections, that represented 18 ABC, 4 unclassified, and 20 GBC DLBCL cases based on COO subtyping by the Affymetrix GeneChip technology (gold standard of classification)^20^. For the two validation cohorts, which comprised 42 and 31 DLBCL cases, respectively, COO classification had been accomplished by NanoString nCounter gene-expression profiling using the most recent version of the Regensburg classifier^7^, with feature weights and thresholds given in Szczepanowski *et al*.^21^. The second validation cohort (n = 31) included only DLBCL cases aged >75 years at the time of diagnosis.

### Tissue lysis and protein extraction

Following an established, slightly modified protocol^17,22^, 10-µm FFPE tissue sections were first deparaffinized twice in 1.8 mL xylene at 56 °C for 30 min each, then rehydrated at room temperature with a graded ethanol series of 100%, 85% and 75% for 5 min each, before the sections were dried in an Eppendorf (Wesseling, Germany) speed vacuum concentrator. The dried sections were weighed (9.1 ± 2.0 mg of tissue) and a 20-fold excess of lysis buffer (20 mM Tris-HCl, pH 8.8, 200 mM glycine, 200 mM DTT, 4% SDS) was added to each section. Following sonication for 15 min in an ultrasonic bath, the samples were incubated under continuous agitation first at 99 °C for 30 min, then at 80 °C for 60 min. After the samples had cooled down to room temperatures, they were centrifuged at 12,000 × g for 10 min. Protein content of the pellets was determined by the FluoroProfile kit (Sigma-Aldrich, Taufkirchen, Germany). Protein amounts of up to 400 µg were obtained per tissue section.

### Gel-assisted sample preparation

Gel-assisted tryptic digestion of the extracted proteins was performed according to Fischer and Kessler^23^. Briefly, 50 µg of extracted protein were diluted to a concentration of 1 µg/µL in GASP buffer (50 mM Tris-HCl, pH 8.8, 6 M urea, 1.5 M thiourea, 4% SDS), followed by the addition of 50 µL of a 40% acrylamide/bis-acrylamide solution (AppliChem Inc., Omaha, NE, USA), whereupon the thiol group of the cysteine residues of the extracted proteins reacted with acrylamide to form cysteine-S-ß-propionamide, thus replacing the otherwise customary alkylation of cysteine residues with iodoacetamide. Polymerization was initiated by the addition of 5 µL each of TEMED (Carl Roth GmbH&Co.KG, Karlsruhe, Germany) and 10% ammonium persulfate (Sigma-Aldrich, Munich, Germany). The resulting gel block was then shredded by centrifugation at 12,000 × g for 2 min through a Corning Costar Spin-X centrifuge tube filter without membrane (CLS9301, Sigma-Aldrich). The gel pieces were fixed in 500 µL ethanol/acetic acid/water (40/10/50) for 15 min at room temperature followed by the addition of 1 mL of acetonitrile (LC-MS grade, VWR International Ltd., Lutterworth, UK). After 10 min, the supernatant was discarded and the fixed gel pieces were washed three more times by the consecutive addition of 500 µL of 50 mM ammonium bicarbonate buffer and 1 mL of acetonitrile under constant agitation (400 rpm). The washed gel pieces were dried in a speed vacuum concentrator, before proteins were digested overnight in 100 µL of 50 mM ammonium bicarbonate buffer upon addition of 1.25 µg mass spectrometry approved trypsin (Serva, Heidelberg, Germany). Subsequently, for peptide extraction, 100 µL of acetontrile were added. After 15 min of incubation under constant agitation (400 rpm), the supernatant was transferred to a new 1.7-mL maximum recovery Axygen centrifuge tube (Kinesis GmbH, Langenfeld, Germany). The gel pieces were then incubated once more for 10 min in 100 µL of 5% formic acid (LC-MS grade, VWR), followed by the addition of an equal volume of acetontrile for 10 min. The supernatants were combined and dried in a speed vacuum concentrator.

For LC-SWATH-MS, the dried tryptic peptides were dissolved in 50 µL of 5% formic acid, containing in addition 50 fmol (for nanoLC; 100 fmol for microLC) of the retention time standard RePLiCal (PolyQuant GmbH, Bad Abbach, Germany), which comprises 27 peptides that cover the entire LC gradient for normalization of retention times by linear fitting.

### Proteome analysis by nanoLC-MS/MS on an AB Sciex TripleTOF 5600+ system

An aliquot of 0.5 µg of digest was used for nanoLC-MS analyses. For the generation of the peptide library, the 20 GCB cases (pool 1) as well as the 18 ABC and 4 unclassified cases (pool 2) of the training set were pooled and each of the two pools was then subjected to 6 runs of information-dependent acquisition (IDA). Separation of tryptic peptides was performed on a Dionex Ultimate3000 nano-HPLC (ThermoFisher, Dreieich, Germany) using peptide trapping. Therefore, the tryptic peptides were trapped at 45 °C on an Acclaim PepMap trapping column (5 mm × 300 µm i.d., 5-µm particle size) using 0.1% formic acid in deionized water (PureLab Plus system, ELGA LabWater, Celle, Germany) at a flow rate of 5 µL/min. Separation was accomplished by a 180 min-binary acetonitrile (LC-MS grade, VWR) gradient with 0.1% formic acid (3–40% in 180 min) on a 25-cm Acclaim (PepMap column, 75 µm i.d., 3 µm particle size, flow rate of 300 nL/min at 45 °C). For peptide library generation, the TripleTOF 5600+ mass spectrometer (AB Sciex, Darmstadt, Germany) was operated in IDA mode from 400–1,000 *m/z* for 250 ms, followed by MS/MS-spectra from 230–1,500 *m/z* of the 30 most intensive precursor ions for 100 ms per precursor. The data were searched using ProteinPilot 5.0 (AB Sciex) against the UniProtKB/Swiss-Prot (December 2016) database with the following adjustments: sample type “Identification”, Cys alkylation “Acrylamide”, digestion “Trypsin”, taxonomy “*Homo sapiens*”, search effort “RapidID”, special factors “Gel-based ID” and detected protein threshold “0.05 (10%)” with false discovery rate (FDR) analysis.

For subsequent SWATH-analyses, the same chromatographic conditions as for the IDA-runs were used. Given a typical peak width (base-to-base) of more than 30 s, at least seven data points were obtained per LC peak using a fixed duty cycle length of 4.5 s. After a 50 ms TOF-MS scan, the entire *m/z* range of 230–1,500 was covered using 60 SWATH windows of 75 ms each and variable quadrupole isolation width (7–25 *m/z*)^24,25^. Targeted extraction of proteins from the SWATH runs was accomplished by the SWATH Acquisition MicroApp 2.0.1 within the PeakView 2.2 software (AB Sciex). Quantification by the PeakView 2.2 software was based on the six most abundant, proteotypic peptides per protein group (≥8 amino acids in length, six transitions per peptide) with an FDR < 1% and an identification confidence >95% using a ± 5-min retention time window after retention time alignment. Quantification was subsequently restricted to proteins that had less than four missing values (as judged by an FDR < 1% on the peptide level) over the combined development and first validation cohort, thus ensuring that only the most reproducible peptide spectra were used for model training. Protein intensities were normalized by the total sum of protein intensities of the respective sample. The mass spectrometry data has been deposited to the ProteomeXchange Consortium via the PRIDE partner repository with the dataset identifier PXD012317^26^.

### Proteome analysis by microLC-MS/MS on an AB Sciex TripleTOF 5600+ system

MicroLC separations were carried on an ekspert nano/microLC 425 system (AB Sciex) coupled to a TripleTOF 5600+ mass spectrometer via the DuoSpray source. Ten µL of tryptic digest (5 µg) were injected directly onto a 150 × 0.3 mm I.D. YMC-Triart C18 column (particle size 1.9 µm, 120 Å, YMC Europe GmbH, Dinslaken, Germany) and peptides were separated at a flow rate of 6 µL/min and a column temperature of 40 °C with a 100-min linear acetonitrile gradient (2–40%) in 0.1% formic acid. SWATH-MS parameters were identical to those listed above for nanoLC-SWATH-MS. Using the peptides of the RePLiCal standard added to the samples, retention times of the peptides detected were adjusted to those of the peptide library. Overall, approximately 90% of the peptides that had been identified by information-dependent nanoLC-MS/MS could be quantified by microLC-SWATH-MS.

### Targeted analysis of the protein signature by selected reaction monitoring

Samples were injected into an UltiMate 3000 RSLCnano HPLC system (Thermo Fisher Scientific, Waltham, MA, USA) using a 1.0 µL-pickup injection (0.5 µg of peptide digest). At a flow rate of 10 µL/min and using a mobile phase of 2.0% acetonitrile (Sigma Aldrich, St. Louis, MO, USA) with 0.05% (v/v) trifluoroacetic acid (Sigma Aldrich) in water, peptides were loaded onto an 5.0 × 0.3 mm i.d. Acclaim™ PepMap™ 100 C18 trap cartridge (Thermo Fisher Scientific). After 10.0 min, peptides were eluted onto a 200 × 0.1 mm i.d. capillary column packed in-house with 3.0-µm Hypersil GOLD™ aQ C18 particles for peptide separation. Water (A) and acetonitrile (B) with 0.1% (v/v) formic acid (FA, Sigma Aldrich) were used as eluents. For the separation, the column temperature was set to 50 °C and a linear gradient of 6–40% B in 120 min at a flow rate of 0.5 µL/min was used. The HPLC-system was hyphenated to a TSQ Vantage™ triple-stage quadrupole mass spectrometer (Thermo Scientific) by a nano-electrospray ionization source. The mass spectrometer was operated in selected reaction monitoring (SRM) mode with positive ionization. The capillary temperature was set to 350 °C with a vaporizer temperature of 50 °C and a spray voltage of 1,600 V. Fragmentation was performed with a collision gas pressure of 1.5 mTorr. The three most intensive transitions for each of the preselected peptides were chosen and individual SRM collision energies were set according to PINPOINT 1.0 (Thermo Scientific) in the range of 20 to 41. The cycle time was 2 s with a Q1 peak width of 0.7 FWHM. Data analysis was performed using PINPOINT 1.0. Precursor ions and related transitions were uploaded and processed with a peak width of 1 min and a minimum signal threshold of 50 to calculate file areas.

### Targeted analysis of the protein signature by parallel reaction monitoring

Using an UltiMate 3000 RSLCnano HPLC system (Thermo Fisher Scientific), tryptic peptides were loaded onto an 5.0 × 0.3 mm i.d. Acclaim™ PepMap™ 100 C18 trap cartridge (Thermo Fisher Scientific) using a 1.0 µL-pickup injection (0.5 µg of peptide digest) at a flow rate of 10 µL/min and 2.0% acetonitrile (Sigma Aldrich) with 0.05% (v/v) trifluoroacetic acid (TFA, Sigma Aldrich) in water. After 10 min, peptides were eluted onto a 200 × 0.1 mm i.d. capillary column packed in house with 3.0-µm Hypersil GOLD™ aQ C18 particles for separation. Water (A) and acetonitrile (B) with 0.1% (v/v) formic acid (Sigma Aldrich) were used as eluents. The column temperature was set to 50 °C and a linear gradient of 6–30% B in 80 min followed by 30–45% B in 60 min at a flow rate of 0.5 µL/min was used for the separation of peptides. The HPLC system was hyphenated to a Q Exactive™ Hybrid Quadrupole-Orbitrap™ mass spectrometer (Thermo Scientific) by means of a nano-electrospray ionization source. The source was operated in positive ion mode with a spray voltage of 1,400 V. For parallel reaction monitoring (PRM) analysis, MS^2^ scans of target ions from an inclusion list were recorded with a resolution of 17,500 (at *m/z* 200). The AGC target was 1e^5^ with a maximum injection time of 50 ms. Target ions were isolated with an isolation window of 2.0 *m/z* and fragmented with a normalized collision energy (NCE) of 29.

### Model training and validation

For classification of DLBCLs into their COO subtypes, we trained a penalized linear regression model on the 780 protein groups of the training set that had been also successfully quantitated in the first validation set. For this purpose, we performed zero-sum elastic net regression^18,19^, with GCB scores provided by the MMML consortium^21^, as the continuous response variable and protein intensities as predictor variables. Since zero-sum regression does not require any prior normalization steps, we used the raw, log_2_-transformed protein group intensities as predictor variables. Furthermore, we set the parameter $\alpha$ to 1, which corresponds to penalizing the protein weights by the l_1_ norm. This choice produces linear models sparse in the number of proteins^27^.The penalizing parameter $\lambda$, which controls overfitting, was calibrated in a tenfold cross-validation. For the final model, we employed ‘lamdba.1se’ for $\lambda$ as proposed by Friedman *et al*.^28^. The decision boundaries, which assign labels ABC/GCB/unclassified to the predicted GCB scores, were trained by minimizing the number of misclassifications.

The application of a zero-sum signatures on data from a different measurement platform requires the adjustment of the offset ${\beta }_{0}$. Here, ${\beta }_{0}$ was estimated as the mean difference between predicted scores on nanoLC-SWATH-MS and microLC-SWATH-MS, nanoLC-SWATH-MS and nanoLC-SRM-MS, and nanoLC-SWATH-MS and nanoLC-PRM-MS, respectively.

The nanoLC-PRM-MS data contained zero entries and missed intensities for protein RPS15. Those values were replaced by the lowest signal intensity in the data matrix prior to log_2_ transformation.

## Results

### Generation of a sparse ABC/GCB classifier

Following an established, albeit slightly modified protocol^17,22^, a single 10-µm thick FFPE tissue section per DLBCL case was first deparaffinized in xylene and rehydrated in ethanol, before proteins were extracted and subjected to gel-assisted tryptic digestion according to Fischer and Kessler^23^. Tryptic peptides were then separated by reversed-phase nano-liquid chromatography and detected by means of a SCIEX TripleTOF 5600+ mass spectrometer using information-dependent acquisition for peptide library construction and data-independent acquisition for label-free quantification. Depending on the ProteinPilot version used to query the annotated human protein sequences deposited in the Swiss-Prot database, 2,702 (version 4.5), respectively 3,210 protein groups (version 5.0) could be identified with a FDR of <1% in the pooled GCB as well as ABC and unclassified cases of the training set. Limiting quantitation to protein groups that could be quantified in at least 39 cases each of the training and first validation cohort, a total of 942 and 888 protein groups, respectively, could be quantified by data-independent SWATH-MS acquisition in the two cohorts, with an overlap of 780 protein groups. The log_2_-transformed signal intensities of these 780 protein groups were then used as independent variables in reference point insensitive zero-sum elastic net regression with variable selection to build a sparse protein signature capable of predicting the Affymetrix GeneChip based GCB scores of the 42 DLBCL cases of the training set. For variable selection, the sparsity parameter $\lambda$ was optimized by tenfold cross-validation using’lambda.1se’ as proposed by Friedman *et al*.^28^. The identities of the eight proteins that constituted the ultimate model and their respective regression coefficients as well as the intercept are given in Table 1. To calculate the GCB score of a new specimen, the log_2_-transformed totals of the signal intensities of the eight proteins are first multiplied with their respective regression weights and then added up together with the intercept. Positive and negative ß-coefficients indicate proteins that are more or less abundant in GCB-like DLBCL, respectively. However, since the regression model is built on ratios rather than absolute signal intensities of the individual proteins, abundances do not necessarily have to differ between GCB- and ABC-like cases. Actually, significant differences in abundance were only observed for periostin (POSTN; ß = +1.55; p = 0.00076) and lumican (LUM; ß = +1.49; p = 0.00058), both of which were upregulated in GCB-like DLBCL, and the immunoglobulin heavy constant mu (IGHM; ß = −2.56; p = 0.002) and the tyrosine-protein phosphatase non-receptor type 1 (PTN1; ß = −3.48; p = 0.00001), both of which were downregulated in GCB-like DLBCL.

**Table 1:** Protein identities, regression weights and intercept of the zero-sum model trained on the development cohort.

UniProt protein ID	Regression weight
P04233 | HG2A_HUMAN	+4.489047570
Q15063 | POSTN_HUMAN	+1.551003753
P51884 | LUM_HUMAN	+1.490571730
Q9NS69 | TOM22_HUMAN	+1.019845999
P62841 | RPS15_HUMAN	−0.888995137
P16070 | CD44_HUMAN	−1.624465232
P01871 | IGHM_HUMAN	−2.556118873
P18031 | PTN1_HUMAN	−3.480889810
Intercept	−1.258554293

**Figure 1:**
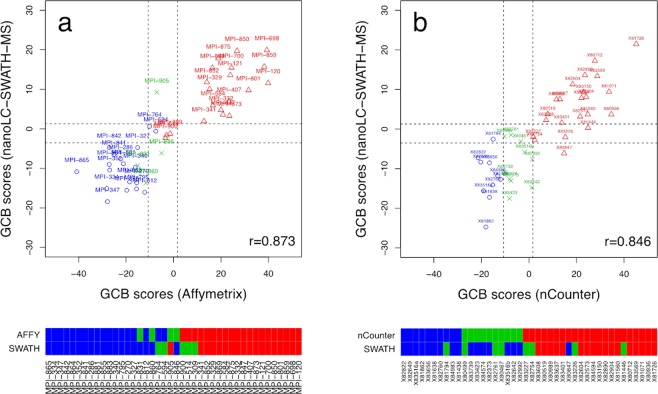
DLBCL subtyping using different methods of gene and protein expression profiling and tissue preservation. Plot (**a**) shows the GCB gold-standard scores based on Affymetrix GeneChip expression profiling of cryo-preserved tissue sections versus zero-sum scores predicted in a leave-one-out cross validation on nanoLC-SWATH-MS proteome data acquired on FFPE sections that had been prepared in parallel for a total of 42 DLBCL cases. The scores from both technologies agree well with a Pearson correlation coefficient of r = 0.873 despite the differently preserved tissue sections used. The dashed lines are classification boundaries for GCB, unclassified and ABC, respectively. The color bars below the plot contrast the respective classifications, with the absence of major disagreements underscoring the good agreement between the Affymetrix and the nanoLC-SWATH-MS based COO assignments. Plot **(b**) shows the correlation between nCounter and nanoLC-SWATH-MS based GCB scores with a Pearson correlation coefficient of r = 0.846 and the respective COO assignments obtained for an independent set of 42 FPPE tissue sections. ABCs are indicated by blue circles, unclassified cases by green crosses, and GCBs by red triangles, respectively.

Figure 1a shows the correlation (r = 0.873) between the nanoLC-SWATH-MS based GCB scores predicted on FFPE tissue versus the gold-standard Affymetrix GeneChip scores obtained on fresh frozen material. The thresholds (dashed lines) for classifying the samples as either ABC, unclassified or GCB were chosen as to minimize the number of misclassifications. The corresponding decision boundaries are −3.50 and 1.28. Specimens with a predicted score lower than −3.50 are labeled as ABC, while specimens with a score larger than 1.28 are labeled as GCB. If a score is in between these boundaries, the specimen is labeled as unclassified. The heat map below the plot contrasts the Affymetrix and nanoLC-SWATH-MS based COO assignments. A total of 9 minor misclassifications were observed, *i.e*., ABCs/GCBs that are labeled as unclassified and *vice versa*. A similarly strong Pearson correlation coefficient of r = 0.846 was obtained for the independent validation cohort of 42 DLBCL specimens, whose GCB scores had been derived from NanoString nCounter gene and nanoLC-SWATH-MS protein profiling of FFPE material (Fig. 1b). We observed 12 minor and a single major misclassification. Compared to the training set, the total number of disagreements had increased from 9/42 (21.4%) to 13/42 (31.0%). The former rate of minor discrepancies is actually quite similar to those observed by Masqué-Soler *et al*.^7^ and Scott *et al*.^10^ for the comparison of GeneChip and nCounter based COO assignments with 16.1% (5 of 31) and 17.9% (12 of 67, including one major disagreement), respectively.

### Reproducibility of GCB scores on microLC-SWATH-MS

**Figure 2:**
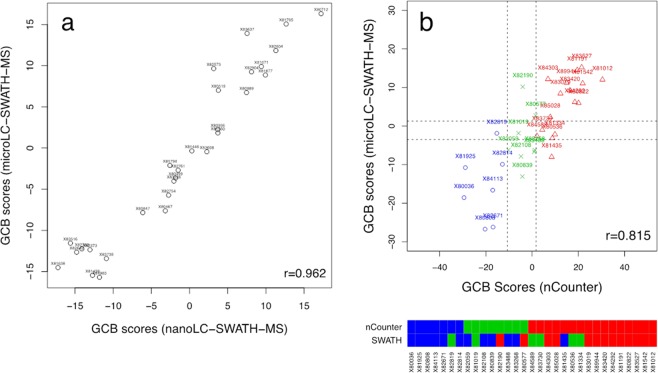
Reproducibility of GCB scores using microLC-SWATH-MS. (**a**) Correlation (r = 0.962, n = 29) between nano- and microLC-SWATH-MS GCB scores based on the summed signal intensities of the 8 proteins constituting the cell-of-origin signature (Table 1). The offset ß_0_ was estimated by calculating the mean difference between nano- and microLC-SWATH-MS GCB scores. (**b**) Correlation (r = 0.815) and respective COO assignments obtained by nCounter and microLC-SWATH-MS based COO subtyping of 31 FFPE tissue sections obtained fromDLBCL patients aged >75 years at the time of diagnosis.

The use of nanoLC and nano-electrospray ionisation offers the advantage that only submicrogram quantities of proteins are required for analysis. However, even FFPE sections as thin as 2 µm yield dozens of micrograms of protein, thus allowing the use of more reliable microLC instrumentation and closed electrospray ionisation sources. Therefore, we explored the feasibility of reproducing GCB scores by microLC-SWATH-MS. As evident from Fig. 2a, after adjustment of the retention times of the peptides contained in the peptide library generated from nanoLC-MS/MS IDA analyses using the RePLiCal standard added to all samples, an excellent agreement of the GCB scores (r = 0.962) was observed.

MicroLC-SWATH-MS was then also used to investigate whether patient age might impact COO classification. To that end, we subjected 31 tissue sections from DLBCL patients aged >75 years to both NanoString nCounter and microLC-SWATH-MS. Despite a fairly good correlation of the GCB scores (r = 0.815), the percentage of minor and major misclassifications had increased to 38.7% (12/31) and 3.2% (1/31), respectively (Fig. 2b). Interestingly, while nanoLC-SWATH-MS assigned roughly equal proportions of ABC- and GCB-like cases across both the training and the two validation sets, which concorded with the Affmetrix GeneChip based COO assignments (Fig. 1a), NanoString nCounter assigned more than twice as many specimens to GCB-like than ABC-like DLBCL.

### Reproducibility of GCB scores on other mass spectrometry platforms

**Figure 3:**
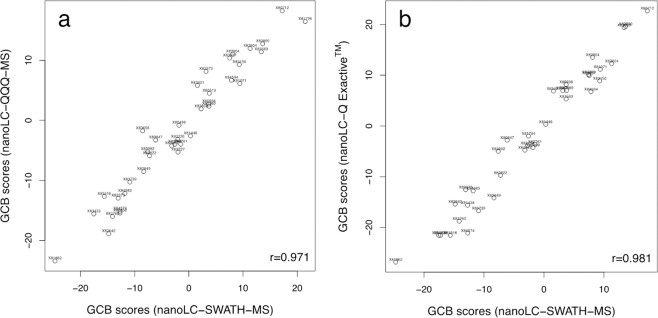
Replication of SWATH-MS GCB scores by targeted analysis of 17 peptides proteotypic for the 8 proteins constituting the cell-of-origin protein signature. (**a**) Plots show the correlation between the nanoLC-SWATH-MS based GCB scores and the GCB scores obtained by the targeted analysis of the 17 proteotypic peptides listed in Table 2 by either (**a**) nanoLC-QQQ-MS (r = 0.971, n = 38) or (**b**) nanoLC Q Exactive™ Hybrid Quadrupole-Orbitrap™ mass spectrometer -MS (r = 0.981, n = 36).

A disadvantage of commercially available gene expression-based technologies, such as nCounter, Illumina’s DASL (for cDNA-mediated Annealing, Selection, extension, and Ligation) platform, and HTG Molecular Diagnostics EdgeSeq system, for COO subtyping in FFPE specimens is the dependence on proprietary instruments and reagents. In contrast, the protein signature derived by zero-sum regression from the nanoLC-SWATH-MS data should be readily transferrable to other platforms for the determination of protein abundance as long as these platforms provide a similar dynamic range^19^. To confirm this, the tryptic digests of 38 DLBCL specimens together with a list of seventeen peptides (Table 2) that had been selected because of their robust detection in FFPE tissue sections, were analyzed independently in the laboratory of Prof. Huber at the University Salzburg, Austria, where the summed signal intensities of each of the eight signature proteins were determined either by selected reaction monitoring of the three most intense fragment ions of each peptide on a triple-stage quadrupole mass spectrometer or by parallel reaction monitoring of the six most abundant trasitions of each peptide on a Q Exactive hybrid quadrupole-Orbitrap mass spectrometer. The offset ${\beta }_{0}$ of the zero-sum signature was adjusted for both monitoring approaches and the GCB scores were then computed as outlined above. Both diagrams in Fig. 3 attest to the excellent concordance of GCB scores regardless of the mass spectrometric method and number of transitions acquired. This appears quite impressive given that no stable isotope-labeled internal standards were used to account for matrix effects that may suppress or increase signal intensities.

**Table 2:** Amino acid sequences, nanoLC retention times, and SWATH-MS precursor and three most intense fragment ions of the 17 proteotypic peptides used for the analysis of the cell-of-origin protein signature by selected and parallel reaction monitoring.

Protein	Peptide sequence	RT (min)	Precursor (m/z^2+^)	Three most intensive fragment ions (m/z^1+^)
IGHM	QIQVSWLR	80	515.2957	788.4413 (y_6_); 561.3143 (y_4_); 660.3828 (y_5_)
IGHM	[PGQ]-QIQVSWLR^a^	114	506.7823	561.3143 (y_4_); 660.3828 (y_5_); 788.4413 (y_6_)
POSTN	AAAITSDILEALGR	116	700.8908	1074.5790 (y_10_); 973.5313 (y_9_); 886.4993 (y_8_)
PTN1	MGLIQTADQLR	82	623.3347	831.4319 (y_7_); 703.3734 (y_6_); 944.5160 (y_8_)
PTN1	FSYLAVIEGAK	100	599.3293	687.4036 (y_7_); 800.4876 (y_8_); 963.5510 (y_9_)
CD44	YGFIEGHVVIPR	82	462.9225	906.5156 (y_8_); 583.3926 (y_5_); 484.3242 (y_4_)
CD44	ALSIGFETC[PPa]R^b^	79	584.2950	783.3454 (y_6_); 983.4615 (y_8_) 726.3239 (y_5_)
CD44	ESSETPDQFMTADETR	71	922.3862	1310.5681 (y_11_); 970.4299 (y_8_); 1411.6158 (y_12_)
HG2A	DLISNNEQLPMLGR	103	800.4116	1258.6208 (y_11_); 814.4604 (y_7_); 1171.5889 (y_10_)
HG2A	LTVTSQNLQLENLR	87	814.9520	1315.6964 (y_11_); 1214.6488 (y_10_); 999.5582 (y_8_)
LUM	FNALQYLR	90	512.7823	763.4461 (y_6_); 579.3250 (y_4_); 692.4090 (y_5_)
LUM	LPSGLPVSLLTLYLDNNK	142	653.0383	766.3730 (y_6_); 864.5189 (b_6_); 980.5048 (y_8_)
RPS15	GVDLDQLLDMSYEQLMQLYSAR	171	863.4172	981.5186 (y_8_); 1109.5771 (y_9_); 1238.6198 (y_10_)
RPS15	GVDLDQLLDM[Oxi]SYEQLMQLYSAR^c^	166	868.7488	981.5186 (y_8_); 1109.5771 (y_9_); 1238.6198 (y_10_)
RPS15	DMIILPEMVGSMVGVYN[Dea]GK^d^	142	685.3384	1012.4768 (y_10_); 737.3611 (b_14_); 734.8442 (y_14_)
TOM22	LQMEQQQQLQQR	50	779.3937	1316.6376 (y_10_); 1056.5544 (y_8_); 1185.5970 (y_9_)
TOM22	LWGLTEMFPER	115	689.8448	1079.5190 (y_9_); 909.4135 (y_7_); 808.3658 (y_6_)

^a^[PGQ]-Q, cyclized N-terminal glutamine; ^b^C[PPA], propionamidated cysteine; ^c^M[Oxi], oxidized methionine; ^d^N[Dea], deamidated asparagine.

## Discussion

Targeted gene expression profiling in FFPE lymphoma tissue sections by NanoString’s nCounter platform has become the method of choice for COO assignment in DLBCL, as various immunohistochemical approaches failed to emulate the predictive power of array-based gene-expression profiling^29^. Major advantages of the nCounter gene expression system are its excellent sensitivity that allows the direct measurement of mRNA expression levels without enzymatic reactions using as little as 200 ng of RNA and its high multiplexing capability. This enables the simultaneous expression profiling of a few hundred different gene transcripts^30^. A potential drawback is the reliance on expensive proprietary instrumentation and reagents.

Analytical approaches based on proteins rather than RNA benefit from the enhanced stability of proteins in FFPE tissue in comparison to nucleic acids^31,32^. Moreover, data-independent acquisition methods like SWATH-MS can yield a comprehensive and quantitative overview of the proteome that can be used subsequently in several ways, e.g., for global differential analyses, extraction of regulation within distinct pathways or identification of potential biomarkers^33^. Such untargeted approaches therefore yield additional data compared to targeted analyses on the RNA-level such as the NanoString nCounter approach, which may be used for additional, more extensive studies.

In contrast to the data-dependent quadrupole Orbitrap tandem mass spectrometric approach taken by Deeb *et al*.^14^, the high scan rates of the here employed TripleToF 5600+ mass spectrometer (25 and up to 100 spectra per second in survey and MS/MS mode, respectively) obviate the need for costly stable-isotope labeled internal standards. Despite the longer duration of the measurements due to lack of multiplexing, the SWATH-technique yields comparable results to stable-isotope labeling strategies^34,35^ and may also complement this data^36^. Fractionation of tryptic digests by strong anion exchange or cation exchange could have been applied to increase the number of peptides. However, we opted to keep the work flow as simple as possible. However, this does not preclude the later extraction of additional peptides from the data-independent SWATH–MS spectra, which represent digital records of all peptides detectable in a given specimen, once expanded peptide libraries become available. Regardless of the technical differences between the present proteomic approach and that employed by Deeb *et al*., it would have been intriguing to test whether the four-protein signature derived by Deeb *et al*. using a support vector machine with t-test based feature selection were to perform equally well as our 8-protein signature. However, this could not be tested as we have succeeded in quantitating to date only three of the proteins, namely TBC1D4, PALD1, and TNFAIP8, but not MME (CD10).

A major difference in the presented bioinformatic method to generate the protein signature is on the one hand the reference-point-insensitivity of the signature making it easily transferable to other instruments and measurement modes. On the other hand the majority of studies use a quantitative proteomics approach to identify significantly regulated proteins and declare the most discriminating features as a potential biomarker signature which is not done in the method presented in this study. Here, all reproducibly quantified proteins are used to generate the signature independent of their regulations. Therefore, the method is based on the ratios between all the proteins within the signature rather than absolute changes. We assumed this method to generate more robust signatures as all signature proteins yield intensive peptide signals in the measurements in contrast to highly regulated proteins which are prone to false assignment of the peptide signals due to absence of the real protein/peptide signals. In fact, only four of the eight protein groups constituting the present biomarker signature would have been designated as “significantly regulated” according to our standard procedures for differential SWATH-MS-based quantification.

Generally, the gene transcripts or protein groups selected for COO subtyping may not necessarily relate directly to differences in biology between ABC- and GCB-like DLBCL. Nevertheless, it appears that the eight protein groups of the present ABC/GCB-classifier and their respective up- or down-regulation in GCB-like DLBCL have some bearing on known biological properties and clinical behavior of DLBCL. For instance, constitutive activation of the NF-κB transcription complex is a key feature of the more aggressive ABC-like subtype^37^. Hence, it is not surprising that CD44, which is a known target gene of NF-κB signaling, exerts a negative influence on the GCB score^38^. Positivity for CD44 expression is predominantly detected in non-GCB-like DLBCL and it is known to be associated with a poor prognosis^39^. The same holds true for PTN1 (PTP1B), whose promoter region does contain a binding site for NF-κB^40^ and whose expression was observed to be preferentially elevated in ABC-like DLBCL^13,41^. The relative up-regulation of lumican (LUM), which is a member of the small leucine-rich proteoglycan (SLRP) family, in turn, indicates extracellular matrix deposition, which is associated with a favorable outcome in patients treated with CHOP alone or in combination with rituximab^42^. Moreover, expression of the related SLRP family member biglycan was found to correlate with the expression of CD40 and the amount of infiltrating macrophages as well as CD4 and CD8 positive T-cells, which are predicative of a better prognosis^43^. Periostin (POSTN) is also a matricellular protein that plays an important role in adhesion and migration of cells and remodeling of the extracellular matrix^44^. In cancers and glioblastomas, it has been associated with tumor progression, but little is known about its role in lymphomas. In cutaneous T-cell lymphomas, periostin is expressed mainly in early disease and appears to increase expression of Th1 chemokines, which might attract Th1-dominant antitumor CD8 T cells^45^. Thus, its expression could indicate a positive prognosis as supported here by its relative up-regulation in GCB-like DLBCL. TOM22, which is a core component of the mitochondria outer membrane protein location pore, has been reported to be a mitochondrial receptor for the proapoptotic protein Bax and, thus, to play a role in Bax-dependent apoptosis^46^. Immunostaining of Bax in DLBCL has served as a statistically significant factor in prognosticating both overall (P = 0.0015; 3-year OS rates of 81.7% and 46.3% for Bax-positive and Bax-negative cases, respectively) and disease-free survival (P = 0.0052; 3-year DFS rates of 80.5% and 44.6% for Bax-positive and Bax-negative cases, respectively)^47^. Hence, the relative up-regulation of TOM22 in GCB-like DLBCL, which is more responsive to CHOP or CHOP-like regimens, does not come as a surprise. Loss of expression of major histocompatibility complex class II (MHC II) genes and associated genes such as the HLA-DR antigen-associated invariant chain (HG2A or CD74) has been related to the non-GCB subtype and an inferior overall survival in R-CHOP-treated DLBCL patients due to impaired antigen presentation and immune surveillance^48^. This is in line with the relative up-regulation of CD74 observed here in GCB-like DLBCLs. Relative overexpression of the *IGHM* gene, in contrast, is a key feature of ABC-like DLBCL^49^, and *IGHM* has been part of an 18-gene signature that differentiated ABC- and GBC-like DLBCLs with an overall crossvalidation error of approx. 6%. Finally, ribosomal protein S15 (RPS15), which is a component of the 40 S ribosomal subunit, has been reported to bind to mouse double minute 2 homolog (MDM2) and to inhibit E3 ligase activity, thereby leading to p53 stabilization and cell cycle arrest^50^. Hence, its relative down-regulation in ABC-like DLBCL is expected to reduce p53 stabilization and to increase its degradation, resulting in reduced cell death and accelerated cell proliferation. In this context it is interesting to note that mutations in RPS15 are a hallmark of aggressive relapsing chronic lympocytic leukemia and have been shown to result in reduced p53 stabilization^51^.

It would have been desirable to have detailed data, for instance, with regard to the cellular composition of the tissues and their content of extracellular matrix, to gain additional insight into the biological system and also possible enhancements of the analytical process, e.g. higher extraction yields, better reproducibility, enhanced proteome coverage, etc. However, in most cases the respective data is not available to the analytical scientists. Nevertheless, there are a number of reasons that may explain the discrepancies observed between gene and protein expression-based COO subtyping. First, tissue sections used for the different subtyping approaches were not necessarily cut adjacently to each other and, therefore, they might vary significantly in the relative content and type of lymphoma and stroma cells as well as extracellular matrix. Given the sizes of the ß-coefficients of the extracellular matrix proteins lumican and periostin in the 8-protein regression equation, intra-lymphoma variability in extracellular matrix may significantly affect the GCB score. Moreover, gene expression analyses had been conducted in part many years earlier and, therefore, degradation of protein that might have occurred in the meantime due to the presence of endogenous or exogenous water cannot be excluded entirely^52^. Nor can the mislabeling of specimens be ruled out. Overall, however, the rate of discrepancies observed between GeneChip based COO classification using cryopreserved tissue and COO assignments made by nCounter and LC-SWATH-MS, respectively, employing FFPE tissue are very similar with minor discrepancies being observed in 1 out of 5–6 cases, while major misclassifications are only found 1–2%^7,10^.

We have shown previously that by forcing the ß-coefficients of elastic net regression to sum to zero, molecular signature become transferable across both molecular levels and technologies without significantly affecting the predictive performance. Here, we have demonstrated the excellent correlation of GCB scores obtained by the original nontargeted LC-SWATH-MS approach and the subsequent targeted approaches of a selected number of tryptic peptides unique to the eight proteins using both selected and parallel reaction monitoring on two different types of mass spectrometers, namely a triple quadrupole and a quadrupole-Orbitrap mass spectrometer, respectively. The long measurement durations in nano-HPLC-based separations, high instrumental cost and mere data amount of data-independent acquisition methods often stand against clinical use of these techniques for monitoring of protein signatures. Thus, the advantage of the novel bioinformatics approach lies in the easy transferability of measurement of the proposed protein signature. The classification was independent of the (nano- or micro-)HPLC-separation, the type of mass spectrometer (Q-TOF, Orbitrap, triple quad), the mode of measurement (SWATH, PRM, SRM) and the applied protein amount (as long as all proteins of the signature can be measured within the instruments´ dynamic range). Moreover, successful transfer of the COO signature is not only limited to other mass spectrometers but may also be accomplished, for instance, by dedicated ELISA assays as long as the abundances of the proteins constituting the signature fall within the linear ranges of the analytical method employed. Only ß_0_ will have to be adjusted between the platforms to obtain identical scores. But while multiplex ELISA assays can be readily established in clinical laboratories and offer great throughput, the continued nontargeted data-independent acquisition of protein expression profiles by LC-SWATH-MS offers the great advantage that the spectra may be mined for signatures other than COO subtyping, including prediction of response to targeted treatment or risk of early relapse. To that end, it is recommended to spike samples with unique peptides not to be found in the human proteome that cover the entire gradient so that spectra can be readily aligned even if shifts in retention time were to occur over time.

In conclusion, label-free LC-SWATH-MS in combination with zero-sum elastic net regression allowed the identification of an eight-protein signature for the molecular classification of DLBCL that could be readily transferred to other analytical platforms, thus obviating the dependence on proprietary instrumentation and reagents. Moreover, the additional proteomic data generated by LC-SWATH-MS might be used in further studies beyond the actual DLBCL classification. Overall, LC-SWATH-MS provides a valuable complementary approach to comprehensive gene expression profiling that can be applied to both fresh, cryopreserved and FFPE tissue specimens^17^.
